# Natural procreative technology (NaProTechnology) for infertility: take-home baby rate and clinical outcomes in a 5-year single-center cohort of 1,310 couples

**DOI:** 10.3389/frph.2025.1696679

**Published:** 2025-11-14

**Authors:** José Ignacio Sánchez-Méndez, María Lombarte, Ricardo Abengózar-Muela, Juan Acosta-Díez, Patricia Alonso-Fernández, María Pilar Cañones-Castañón, Olga Calderón-Ruiz, Elena Espinosa-García, Carolina Galocha-Morgado, Jaime Siegrist, Sonsoles Alonso-Salvador

**Affiliations:** 1 Fertilitas Center, Madrid, Spain; 2 Universidad Francisco de Vitoria, Madrid, Spain; 3 Department of Obstetrics and Gynecology, Hospital Universitario La Paz, Madrid, Spain; 4 IdiPaz, Hospital La Paz Institute for Health Research, Madrid, Spain; 5 Department of Obstetrics and Gynecology, Universidad Autónoma de Madrid (UAM), Madrid, Spain

**Keywords:** natural procreative technology, NaProTechnology, infertility treatment, take-home baby rate, fertility restorative medicine, recurrent pregnancy loss, reproductive health, endometriosis

## Abstract

**Introduction:**

Assisted reproductive technologies (ART) are widely used to address infertility; however, they are costly, associated with medical risks, and often yield suboptimal clinical outcomes. Natural Procreative Technology, also known as NaProTechnology (NPT), provides a systematic and integrative approach to infertility by thoroughly identifying and treating underlying medical conditions to restore the couple's natural fertility potential. Despite its promise, real-world data on NPT effectiveness remain limited. The objective of this study is to evaluate the take-home baby rate in a large population of infertile couples treated with NPT and to synthesize findings from previously published studies.

**Methods:**

A retrospective cohort study was conducted involving 1,310 infertile couples treated at a specialized fertility clinic in Spain over a 5-year period. Participants presented with primary or secondary infertility or recurrent pregnancy loss. Clinical data, diagnoses, and outcomes were analyzed, including surgical interventions and treatment duration.

**Results:**

The mean age of women and men was 35.0 (SD 4.4) and 36.9 (SD 5.3) years, respectively. Primary infertility was the most common subtype (73.5%), the median infertility duration was 24 months, and prior ART attempts were recorded in 27.5% of couples. Mean number of diagnoses per couple was 2.5 (SD 1.3). The crude take-home baby rate was 35.3% (*N* = 463). Independent predictors of successful take-home baby included female age, recurrent pregnancy loss as the reason for consultation, duration of infertility, and the presence of endometriosis, hormonal dysfunction, male factor, and endometrial disorders as diagnoses. Considering a median duration of NPT of 10.9 months (range 8.1–17.0), the adjusted cumulative take-home baby rate was 62.1%. Rates varied significantly by female age, with higher success observed in younger women: 83.7% at 18–30 years, 53.3% at 36–40 years, and 24.4% over 40 years. A sensitivity analysis was performed to assess the impact of dropout assumptions on cumulative pregnancy rates. Nearly one-third of patients underwent surgery, most commonly hysteroscopy and/or laparoscopy.

**Conclusion:**

In this cohort, NPT was associated with a notably high take-home baby rate in an infertile population with unfavorable prognostic factors, including advanced maternal age, prolonged duration of infertility, or previous failed attempts at conventional ART procedures.

## 1 Introduction

Infertility is a disease of the male and/or female reproductive system defined by the inability to achieve pregnancy after 12 months or more of regular sexual intercourse without the use of contraceptives (1). In couples where women are older than 35 years, the commonly accepted cut-off point for infertility is 6 months (2). It is estimated that infertility affects approximately 48 million couples worldwide, accounting for about 10% of couples of reproductive age, with geographical variations (3). A systematic review and meta-analysis of global populations from 1990 to 2021 reported a period prevalence of 12-month infertility of 5.0%–34.0% for high-income countries and 1.6%–32.0% for low-income countries (4). Data from the US National Survey of Family Growth (2015–2019) indicated that 13.4% of women aged 15–49 had impaired fecundity and 11.4% of men experienced some form of infertility; infertility among married women increased from 14.3% in those aged 25–29 years to 26.7% in those aged 35–39 years (5).

In recent decades, assisted reproductive technologies (ART) which involve the manipulation of oocytes and/or embryos, have been widely used to treat infertility (6). In Spain, according to the most recent fertility survey conducted in 2018, 5.4% of women between 18 and 55 years had undergone ART (7), and 12% of births were achieved through ART according to the latest data from the Spanish Society of Fertility (8). By comparison, ART-conceived infants represented approximately 2% of birth in the United States in 2018 (9). However, despite these improvements, ART still disproportionally contributes to multiple births, low birthweight and preterm birth (9). Moreover, the use of ART is associated with increased risks of major non-chromosomal birth defects and greater risk for cancer (10). Multiple gestations have also been suggested as a contributing factor of poor birth outcomes and adverse obstetric morbidity, such as hypertensive disorders of pregnancy and gestational diabetes (11).

To minimize the risks associated with ART for both mothers and offspring, Natural Procreative Technology (NaProTechnology or NPT)—developed by Dr. Thomas Hilgers at the Saint Paul VI Institute for the Study of Human Reproduction in Omaha, NE- provides a systematic and integrative approach to infertility, grounded in detailed evaluation of physical, biochemical, and ultrasound (US) biomarkers of the menstrual cycle (12, 13).

NPT employs the *Creighton Model FertilityCare System* (CrMS) to enable early identification and targeted treatment of underlying conditions contributing to infertility (14, 15), with the primary objective of restoring physiological function to facilitate natural conception. Therapeutic strategies include cervical mucus enhancers, ovulation inducers, hormonal supplementation, antibiotics, immunomodulatory therapies, and other individualized interventions. Clinical management is guided by meticulous menstrual cycle tracking, blood tests, and US imaging, allowing for highly individualized treatment plans (16). While medical management of NPT is often sufficient to achieve successful conception, surgical intervention may be necessary in selected cases.

However, clinical experience with NPT in the management of infertility remains limited. In an Irish general practice, the cumulative proportion of first live births after up to 24 months of NPT treatment was 52.8 per 100 couples (14). In two outpatient clinics in Ireland, the overall live birth rate was 32.1% in 403 women with a history of infertility and prior *in vitro* fertilization (IVF) treatment (17). In a retrospective cohort study con in a Canadian family practice setting, 65.7% of 108 couples achieved live births within 2 years with NPT and all of which were singleton births (18). In two family clinics in Massachusetts, the cumulative live birth rate at 2 years was 29,0% overall among 370 couples, increasing to 34,0% for women under the age of 35 (19). In a multicenter study conducted across 10 clinical sites in four countries, involving 843 subfertile couples, the rate of live birth over a three-year follow-up period was 44.2% (20).

Taken together, these studies provide evidence supporting the promising role of NPT in the management and treatment of couples diagnosed with infertility who wish to have children. The present study, which evaluates the take-home baby (THB) rate in 1,310 infertile couples treated at a specialized fertility clinic in Spain over a 5-year period, represents the largest real-world experience with NPT reported to date—and the first documented in our country. A synthesis of the current findings, alongside data from previous studies retrieved from the literature, provides updated and clinically relevant information on the role of NPT in infertility treatment.

## 2 Materials and methods

### 2.1 Study design and setting

This was a large, retrospective, single-center cohort study conducted in an NPT specialized clinic in Madrid, Spain, which serves patients from across the country.

### 2.2 Participants

The study included couples diagnosed with primary or secondary infertility or recurrent pregnancy loss who received NPT treatment between November 2018 and December 2023 were included. Eligible participants were men and women aged 18 years or older who sought consultation for infertility, regardless of prior fertility treatments.

### 2.3 Exclusion criteria

Patients were excluded if they did not complete the initial NPT evaluation period of 3–6 months or were considered unsuitable for NPT due to limiting medical factors, such as confirmed and irreversible azoospermia, premature ovarian failure, absence of a uterus or bilateral tubal factor not amenable to surgical repair. These exclusions were based on clinical judgment and standardized internal protocols, although detailed documentation of exclusion rationale was not systematically recorded.

No formal exclusion was applied based on infertility duration, type, or previous ART attempts, in order to reflect real-world clinical practice. However, this approach resulted in a heterogeneous population, which is acknowledged as a limitation in the interpretation of subgroup outcomes.

### 2.4 NPT process

All couples were managed by physicians certified in NPT by the Saint Paul VI Institute (Omaha, NE, USA). The NPT process comprises three phases:

**Learning Phase**: Couples received guided instruction from certified CrMS instructors to learn how to observe and chart biomarkers of vaginal secretions, with systematic documentation of this information. Accurate menstrual cycle charting was emphasized as essential for evaluating cycle health.
**Diagnostic Phase**: Based on the interpretation of CrMS observations and biomarker records, together with information obtained from a comprehensive medical history, serial laboratory tests and imaging studies were scheduled. These evaluations enabled the identification of clinical abnormalities and guided the selection of additional diagnostic procedures and individualized therapeutic planning. This phase typically lasted 3–6 months.
**Therapeutic Phase**: Individualized treatment plans were implemented according to the diagnostic findings, including medical and surgical interventions aimed at restoring optimal physiological conditions for natural conception. Once abnormalities were addressed and cycles optimized, pregnancy was expected to occur within 6–12 months. Couples were therefore advised to remain in the NPT program for up to 18–24 months. Standard prenatal care—including scheduled visits, specific blood tests, and US examinations—was provided to women who achieved pregnancy.

### 2.5 Diagnostic evaluation

Once patients were able to accurately identify their fertility biomarkers, a series of evaluations was performed, including both laboratory tests (plasma estradiol and progesterone levels) and US assessments performed over at least one complete menstrual cycle, covering both the follicular and luteal phases to ensure a comprehensive evaluation. At the same time, a basic infertility workup was performed using imaging studies and laboratory tests tailored to each patient's clinical profile.

Diagnoses were grouped into eight categories: functional disorders, including all hormonal abnormalities; endometrial disorders, encompassing both endometritis and morphological abnormalities; male factor; cervical mucus abnormalities; endometriosis; tubal factor; general conditions, referring to systemic or non-reproductive disorders affecting fertility; and other causes, for less common or unclassified findings.

### 2.6 Therapeutic approach

Following the identification of the underlying causes of infertility, the most appropriate interventions were determined to restore optimal physiological conditions for natural conception.

For women, therapeutic measures included medical treatments aimed at regulating hormonal levels (e.g., luteal phase support with progesterone), inducing ovulation (e.g., with gonadotropins, clomiphene citrate or letrozole), enhancing cervical mucus quality (e.g., guaifenesin, or vitamin B6), and anti-inflammatory or antibiotic treatments when clinically indicated. Surgical and microsurgical procedures —such as laparoscopy, hysteroscopy, tubal repair— were performed, when necessary, to restore normal uterine, tubal and/or ovarian anatomy, with systematic implementation of adhesion prevention strategies.

Similarly, for men, a multidisciplinary team comprising urologists and andrologists conducted a comprehensive diagnostic protocol. This included semen analysis and, when necessary, additional assessments such as sperm DNA fragmentation testing or testicular biopsy. Based on the findings, appropriate medical or surgical treatments were provided.

A multidisciplinary approach was also adopted for both male and female patients when clinically indicated, involving specialists such as gastroenterologists, immunologists, endocrinologists, psychiatrists, nutritionists, and psychologists.

### 2.7 Data collection and outcomes

Study data were collected from anonymized electronic health records. Life tables were used to calculate the cumulative THB rate from the start of the couple's evaluation to the date of the last menstrual period of the conception cycle.

The primary outcome was the THB rate observed in the study population. The THB rate was defined as the rate of couples who achieved a live birth resulting in a newborn discharged home. This outcome excludes pregnancies ending in miscarriage, stillbirth, or neonatal death prior to hospital discharge, and is considered a clinically meaningful measure of reproductive success from the patient's perspective. Secondary outcomes included the adjusted THB rate according to duration of participation in the NPT program, factors associated with successful pregnancy and the withdrawal rate.

### 2.8 Time-to-event analysis

For the estimation of cumulative THB rates, time zero was defined as the date of enrolment into the NPT program, corresponding to the start of the diagnostic phase. This point marked the beginning of clinical evaluation and therapeutic planning. The date of the last menstrual period (LMP) was used exclusively to identify the conception cycle for couples who achieved pregnancy and was not used as the starting point for survival analysis.

Right-censoring was applied at the earliest of the following events: transition to ART, loss to follow-up, or the end of the study period (June 30, 2024). These censoring criteria were implemented to maintain consistency in the Kaplan–Meier survival framework and to reduce potential bias arising from informative censoring. However, due to the retrospective nature of the dataset, certain time-to-event variables were not systematically recorded. While the date of enrollment and the date of conception (for those who achieved pregnancy) were available, the exact timing of transition to ART was frequently missing or undocumented. In many cases, patients discontinued NPT without formally notifying the clinic, making it impossible to determine whether ART was initiated. As a result, censoring events could not be reliably classified, and the application of competing risks models (e.g., Fine–Gray or Aalen–Johansen) was not feasible.

### 2.9 Statistical analysis

Descriptive statistics were used to summarize baseline characteristics. Categorical variables were expressed as frequencies and percentages, and continuous variables as mean **±** standard deviation (SD) or median with interquartile range (IQR, 25th–75th percentile), according to the distribution assessed using the Kolmogorov–Smirnov test.

Comparisons between categorical variables were performed using the chi-square test or Fisher's exact test, as appropriate, and comparisons between continuous variables were conducted using the Student's *t-*test or the Mann–Whitney *U* test depending on the distribution. Multivariate analysis was performed using binary logistic regression to identify independent predictors of THB outcomes. Results are reported as odds ratios (ORs) with 95% confidence intervals (CIs). Variables included in the model were selected based on clinical relevance and statistical significance in univariate analysis (*p* < 0.05). Collinearity among covariates was assessed using variance inflation factors (VIF), and no significant multicollinearity was detected. Due to the exploratory nature of the study, no formal correction for multiple comparisons (e.g., Bonferroni) was applied. However, the number of predictors was limited to reduce the risk of overfitting.

Cumulative THB rates were estimated using Kaplan–Meier survival analysis, with time measured from the start of the NPT evaluation to the last menstrual period of the conception cycle. Kaplan–Meier curves were stratified by maternal age (<35, 35–39, ≥40 years). 95% confidence intervals were calculated using Greenwood's formula. Differences between groups were evaluated using the log-rank test. Statistical significance was set at *p* < 0.05. All data were analyzed using the IBM Statistical Package for the Social Sciences (SPSS) version 28.0 (IBM Corp., Armonk, NY, USA).

### 2.10 Ethical considerations

The study was approved by the Ethics Committee of Universidad Francisco de Vitoria (Madrid, Spain), under protocol number 01/2025, dated April 8, 2025. All procedures were conducted in accordance with the principles of the Declaration of Helsinki.

## 3 Results

### 3.1 Characteristics of the study population

A total of 1,310 couples met the inclusion criteria. Baseline characteristics are summarized in Table 1. The mean age was 35.0 years (SD 4.4) for women and 36.9 years (SD 5.3) for men. Primary infertility was the most frequent subtype, observed in 73.5% of participants. The median duration of infertility was 24 months, and prior attempts at ART were reported in 27.5% of cases.

**Table 1:** Baseline characteristics and differences in take-home baby rates according to demographic and clinical variables in 1,310 couples undergoing NaProTechnology for the treatment of infertility.

Variables	Total (*N* = 1,310)	Take-home baby		
		Yes	No	*P* value
Age, years	mean (±SD)			
Women	35.0 (4.4)	33.4 (4.0)	35.9 (4.4)	<0.001
Men	36.9 (5.3)	35.7 (5.1)	37.5 (5.3)	<0.001
Type of infertility	*n* (%)			
Primary	963 (73.5%)	328 (34.1)	655 (68.0)	<0.001
Secondary	223 (17.0%)	81 (36.3)	142 (63.7)	
Recurrent pregnancy loss	104 (7.9%)	54 (51.9)	50 (48.1)	
Prior ART	*n* (%)			
Yes (*n* = 360)	360 (27.5%)	91 (25.3)	269 (74.7)	<0.001
No (*n* = 950)	950 (72.5%)	372 (39.2)	578 (60.8)	
Diagnosis of infertility	*n* (%)			
Functional disorders	1,121 (85.6%)	375 (33.5)	746 (66.5)	<0.001
Endometrial disorders	479 (36.6%)	151 (31.5)	328 (68.5)	<0.02
Male factor	455 (34.7%)	138 (30.3)	317 (69.7)	<0.003
Abnormalities of cervical mucus	335 (25.6%)	108 (32.2)	227 (67.8)	NS
Endometriosis	312 (23.8%)	87 (27.9)	225 (72.1)	<0.001
General conditions	241 (18.4%)	89 (36.9)	152 (63.1)	NS
Tubal factor	173 (13.2%)	44 (25.4)	129 (74.6)	<0.004
Other conditions	96 (7.3%)	23 (24.0)	73 (76.1)	<0.02
	mean (±SD)			
Number of diagnoses per couple	2.5 (1.3)	2.1 (1.3)	2.6 (1.1)	<0.001
	median (IQR)			
Duration of infertility, months	24.0 (12.0–36.0)	18.0 (12.0-)	24.0 (15.0-)	<0.001
Time to surgery, months	6.0 (3.4–11.5)	4.1 (1.8–10.2)	7.2 (4.0–12.1)	<0.005

SD, standard deviation; IQR, interquartile range (25th–75th percentile); ART, assisted reproductive technologies; NS, not significant.

In 98.1% (*n* = 1,285) of the patients, at least one cause of infertility was identified, with hormonal disorders being the most prevalent (85.6%), particularly luteal phase defects, which were present in 869 (68.8%) cases. The mean number of diagnoses per couple was 2.5 (SD 1.3).

At least one surgical intervention was performed in 401 (30.6%) women, with hysteroscopy as the most frequent procedure (*n* = 389), combined with laparoscopy in 208 (15.9%). The median time from enrollment in the NPT program to surgery was 6 months.

### 3.2 Pregnancy rate and factors associated with pregnancy

**Figure 1:**
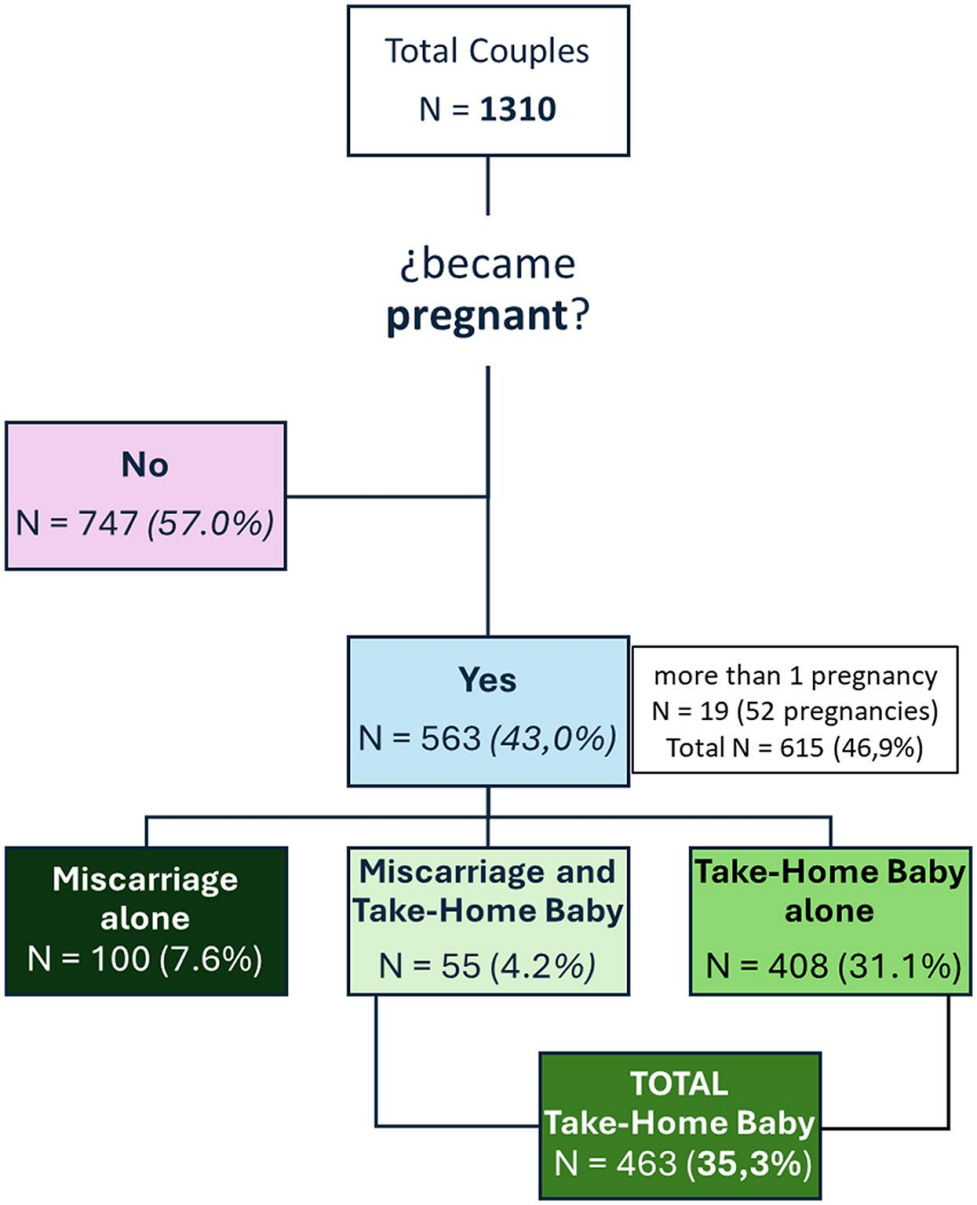
Distribution of patients and achievement of successful pregnancy.

Of the 1,310 couples included in the study, 563 achieved at least one pregnancy, resulting in a crude pregnancy rate of 43.0%. Nineteen women conceived more than once, yielding a total of 615 pregnancies. Among the 1,310 couples, 155 experienced at least one miscarriage, and 55 of them subsequently achieved a successful pregnancy. The overall take-home baby (THB) rate was 35.3%, corresponding to 463 couples (Figure 1). Notably, only one successful pregnancy per couple was considered during the study period, and all primary analyses were conducted at the couple level to ensure consistency in outcome reporting.

As shown in Tables 1, 2 statistically significant differences in the THB rates were observed according to both female and male age, with those who conceived being younger than those who did not. Additional favorable factors included recurrent pregnancy loss as the infertility type, shorter duration of previous infertility, absence of prior ART attempts, a diagnosis of functional disorders, a lower mean number of diagnoses, and a shorter time to surgery. Among the 360 patients with prior ART attempts, one in four (25.3%) achieved a successful pregnancy.

### 3.3 Multivariate analysis

In the multivariate analysis (Table 2) independent predictors of a successful THB included: maternal age between 18 and 30 years (OR: 12.4, 95% CI: 6.6–23.2, *p* < 0.001); consultation for recurrent pregnancy loss (OR: 2.7, 95% CI: 1.7–4.3, *p* < 0.001); a previous infertility duration of 7–12 months (OR: 2.7, 95% CI: 1.9–3.8, *p* < 0.001); and infertility diagnoses such as endometriosis (OR: 1.6), functional disorders (OR: 1.5), male factor (OR: 1.5), and endometrial disorders (OR: 1.4).

**Table 2:** Factors independently associated with take-home baby in the multivariate analysis.

Variable	Odds ratio (95% CI)	*P* value
Women age (years)		
18–30	12.4 (6.6–23.2)	<0.001
31–35	7.7 (4.3–13.8)	<0.001
36–40	4.9 (2.7–8.8)	<0.001
>40 (reference)	1	_
Type of infertility		
Recurrent pregnancy loss	2.7 (1.7–4.3)	<0.001
Infertility (reference)	1	_
Duration of infertility (months)		
1–6	2.6 (1.5–4.5)	<0.001
7–12	2.7 (1.9–3.8)	<0.001
13–18	2.0 (1.4–3.0)	<0.001
19–24	1.6 (1.1–2.3)	<0.016
25–30	1.3 (0.8–2.3)	0.29
>30 (reference)	1	_
Diagnosis of infertility		
Endometriosis		
Yes	1.6 (1.2–2.2)	<0.002
No (reference)	1	_
Functional disorders		
Yes	1.5 (1.2–2.2)	0.014
No (reference)	1	_
Male factor		
Yes	1.5 (1.1–1.9)	0.004
No (reference)	1	_
Endometrial disorders		
Yes	1.4 (1.1–1.8)	0.011
No (reference)	1	_

### 3.4 Timeline analysis

The median duration of the NPT process was 10.9 months (range, 8.1–17.0). The distribution of THB rates and withdrawals over this period is shown in Table 3. Overall, 72.4% of successful pregnancies occurred during the first year, with a median time to conception of 7.1 months (range 5.4–8.8). When restricting the analysis to a minimum follow-up of 3 years (2018–2021), which more accurately reflects the full course of the NPT, the distribution over time was 57.8% (*n* = 115) in the first year, 32.7% (*n* = 65) in the second year, and a residual 8.1% (*n* = 16) in the third year.

**Table 3:** Cumulative take-home baby rates, censored cases, and treatment withdrawals stratified by duration of NaProTechnology process.

Time months	Total *N* (%)	Censored		Withdrawals		Take-home baby rate			
		*N* (%)	Cumulative	*N* (%)	Cumulative	*N* (%)	Cumulative (%)		
			*N* (%)		*N* (%)		*N*	Adjusted | 95% CI	
**Total**	**1,310 (100%)**	**847** **(64.7%)**		**544** **(41.5%)**		**463** **(35.3%)**		**62.1%**	**59.6–64.6**
0–3	1,310 (100%)	0	0	0	0	15 (1.1%)	15 (1.1%)	1.1%	1.1–1.1
4–6	1,295 (98.9%)	0	0	0	0	102 (7.8%)	117 (8.9%)	8.9%	8.8–9.0
7–9	1,193 (91.1%)	192 (14.7%)	192 (14.7%)	152 (27.9%)	152 (11.6%)	136 10.4%)	253 (19.3%)	20.2%	19.8–20.6
10–12	865 (66.0%)	215 (16.4%)	**407** **(31.1%)**	128 (23.5%)	**280** **(21.4%)**	82 (6.3%)	**335** **(25.6%)**	**28** **.** **9%**	**27.4** **–** **30.4**
13–15	568 (43.4%)	122 (9.3%)	529 (40.4%)	83 (15.3%)	363 (27.7%)	35 (2.7%)	370 (28.8%)	33.8%	33.1–34.5
16–18	411 (31.4%)	91 (6.9%)	620 (47.3%)	64 (11.8%)	427 (32.6%)	29 (2.2%)	399 (30.5%)	39.0%	38.0–40.0
19–21	291 (22.2%)	65 (5.0%)	685 (52.3%)	38 (7.0%)	465 (35.5%)	29 (2.2%)	428 (32.7%)	45.9%	44.1–47.7
22–24	197 (15.0%)	55 (4.2%)	**740** **(56.5%)**	27 (5.0%)	**492** **(37.6%)**	13 (1.0%)	**441** **(33.7%)**	**50** **.** **0%**	**48.1** **–** **51.9**
25–27	129 (9.8%)	30 (2.3%)	770 (58.8%)	14 (2.6%)	506 (38.6%)	11 (0.8%)	452 (34.5%)	54.8%	51.9–57.7
28–30	88 (6.7%)	26 (2.0%)	796 (60.8%)	14 (2.6%)	520 (39.7%)	9 (0.7%)	461 (35.2%)	60.3%	56.0–64.6
31–33	53 (4.0%)	19 (1.5%)	815 (62.2%)	9 (1.7%)	529 (40.4%)	2 (0.2%)	463 (35.3%)	62.1%	58.8–65.4
34–36	32 (2.4%)	12 (0.9%)	**827** **(63.1%)**	8 (1.5%)	**537** **(41.0%)**	0	**463** **(35.3%)**	**62** **.** **1%**	**58.8** **–** **65.4**
37–39	20 (1.5%)	8 (0.6%)	835 (63.7%)	2 (0.4%)	539 (41.1%)	0	463 (35.3%)	62.1%	58.8–65.4
40–42	12 (0.9%)	3 (0.2%)	838 (64.0%)	2 (0.4%)	541 (41.3%)	0	463 (35.3%)	62.1%	58.8–65.4
43–45	9 (0.7%)	3 (0.2%)	841 (64.2%)	1 (0.2%)	542 (41.4%)	0	463 (35.3%)	62.1%	58.8–65.4
46–48	6 (0.5%)	1 (0.1%)	**842** **(64.3%)**	1 (0.2%)	**543** **(41.5%)**	0	**463** **(35.3%)**	**62** **.** **1%**	**58.8** **–** **65.4**
49–51	5 (0.4%)	3 (0.2%)	845 (64.5%)	1 (0.2%)	544 (41.5%)	0	463 (35.3%)	62.1%	58.8–65.4
52–54	2 (0.2%)	0	845 (64.5%)	0	544 (41.5%)	0	463 (35.3%)	62.1%	58.8–65.4
55–57	2 (0.2%)	1 (0.1%)	846 (64.6%)	0	544 (41.5%)	0	463 (35.3%)	62.1%	58.8–65.4
58–60	1 (0.1%)	0	**846** **(64.6%)**	0	**544** **(41.5%)**	0	**463** **(35.3%)**	**62** **.** **1%**	**58.8** **–** **65.4**
61–63	1 (0.1%)	0	846 (64.6%)	0	544 (41.5%)	0	463 (35.3%)	62.1%	58.8–65.4
>63	1 (0.1%)	1 (0.1%)	847 (64.7%)	0	544 (41.5%)	0	463 (35.3%)	62.1%	58.8–65.4

CI, confidence interval.

**Figure 2:**
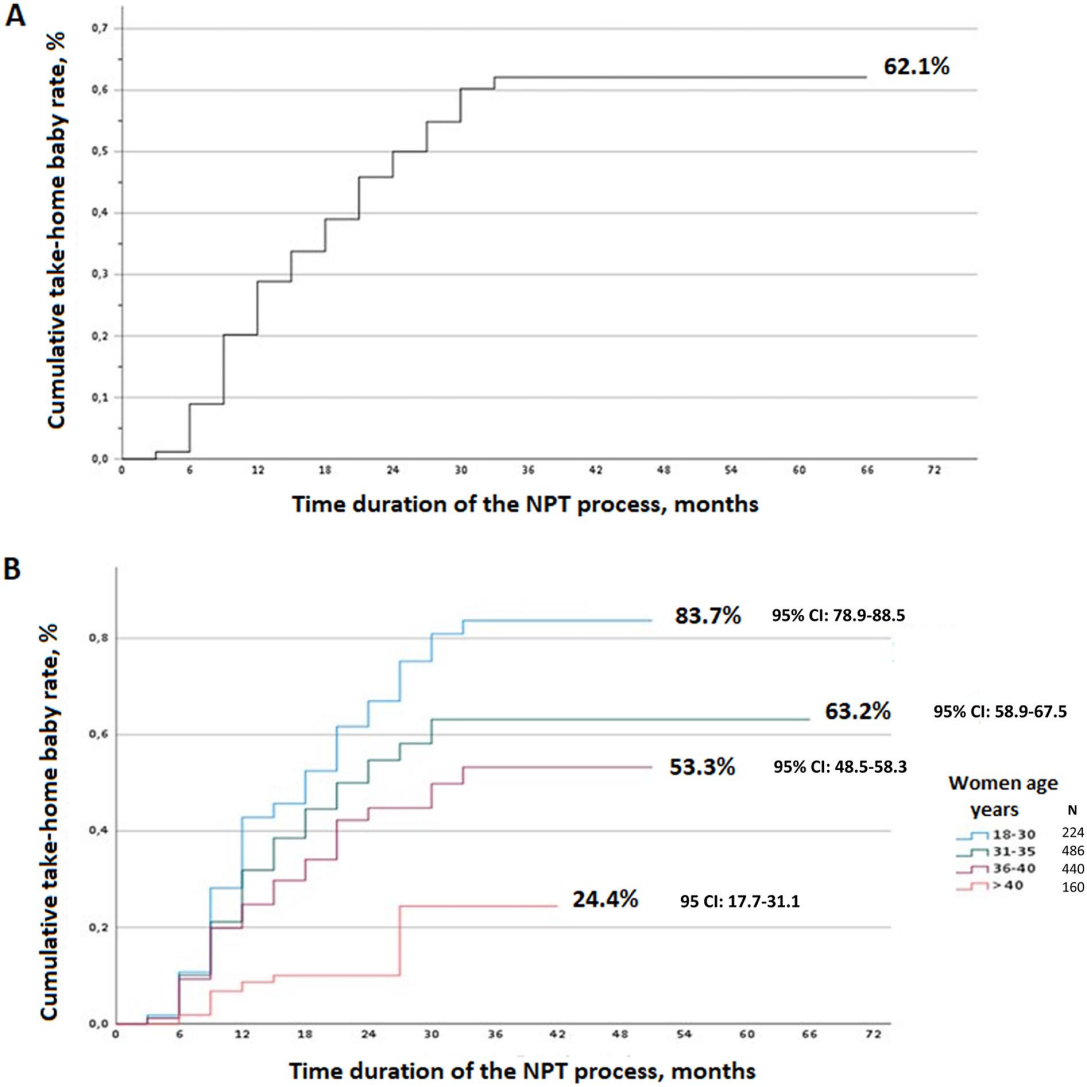
**(A)** Adjusted cumulative take-home baby rate for the overall study population. **(B)** Adjusted cumulative take-home baby rate according to the maternal age.

The adjusted cumulative THB rate was 62.1% (CI: 95%: 58.8–65.4) (Figure 2A). These rates varied significantly (*p* < 0.001) according to maternal age, with higher success observed in younger women: 83.7% at 18–30 years, 63.2% at 31–35 years, 53.3% at 36–40 years, and 24.4% in those over 40 years (Figure 2B).

**Figure 3:**
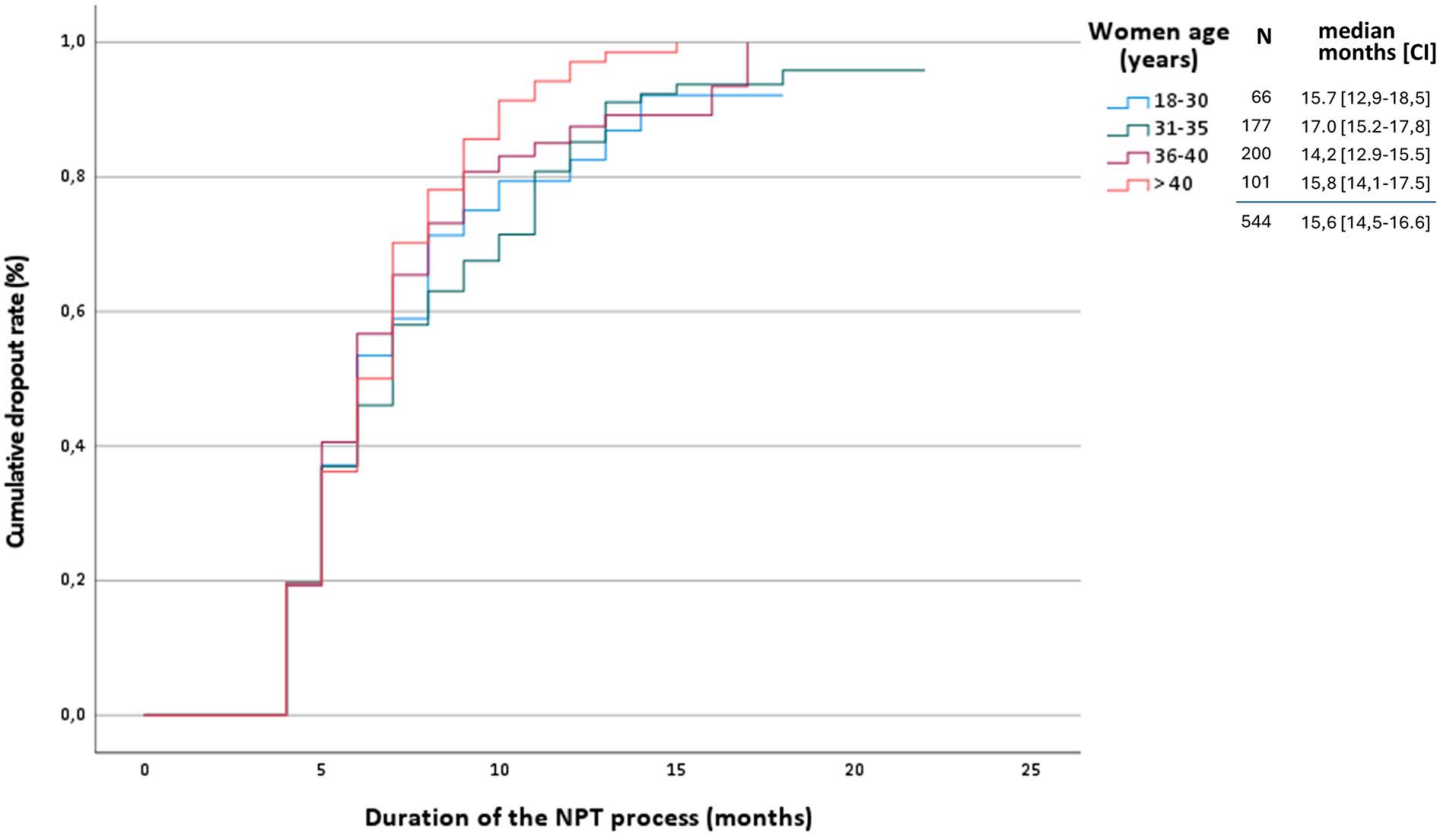
Kaplan–Meier curves showing time to treatment discontinuation, stratified by maternal age groups.

In NPT, the duration of the process is a key determinant outcomes. The overall censored rate was 64.7%, with 31.1% of cases occurring during the first year and 56.5% within the first two years. The cumulative withdrawal rate reached 41.5%, with 21.4% of couples discontinuing treatment during the first year and 37.6% by the end of the second year. The main reasons for withdrawal in the first year were discouragement (31.5%) and transition to ART (17.1%), while treatment completion accounted for the majority of withdrawals in the second year (41.4%). To further explore patterns of treatment discontinuation, we generated cumulative attrition curves stratified by maternal age (Figure 3).

### 3.5 Complications

Although complication rates were low and not systematically recorded, no major adverse events were reported during the study period. Future studies should include detailed surgical outcomes and safety profiles.

## 4 Discussion

A review of the literature identified five previous studies on NPT addressing similar clinical questions (14, 17–20). While Table 4 summarizes their main findings alongside our results, it is important to acknowledge the methodological heterogeneity across these investigations. Most studies were retrospective and observational in nature, with varying sample sizes ranging from 108 to over 1,000 couples. Inclusion criteria differed substantially, particularly regarding prior ART exposure and infertility subtypes, which may influence outcome comparability. Follow-up durations were inconsistently reported, and definitions of pregnancy outcomes (e.g., live birth vs. THB) were not standardized. Furthermore, only a few studies included surgical interventions as part of the NPT process, which limits the generalizability of their findings to comprehensive NPT programs. These differences underscore the need for cautious interpretation of cross-study comparisons and highlight the importance of future multicenter, prospective research using harmonized protocols and outcome measures.

**Table 4:** Main findings of studies of NaProTechnology for infertility published in the literature.

Variable	First author, year					
	Stanford 2008 (14)	Tham 2012 (18)	Boyle 2018 (17)	Stanford 2021 (19)	Stanford 2022 (20)	Sánchez-Méndez 2025
Study period	1998–2002	2000–2006	2004–2010	1989–2014	2006–2016	2019–2023
Number of couples	1,072	108	403	370	834	1,310
Women age (years), median	35.8	35.4	37.2	34.8	34.0	35.3
Duration of infertility (months), median	67	27	69	32	NR	24
Previous recurrent pregnancy loss	11.6%	18.0%	NR	5.9%	7.7%	7.9%
Secondary infertility	24.0%	20.4%	21.8%	26.8%	51.6%	17.0%
Prior ART procedures	33.0%	30.5%	100%	11.8%	21.4%	27.5%
Surgical procedures	NR	11.1%	0	47.6%	21.9%	30.6%
Unexplained infertility	0.5%	0.9%	NR	0.5%	3.9%	1.9%
Crude pregnancy rate	25.5%	38.0%	18.4%	17.8%	44.2%	35.3%
Adjusted pregnancy rate	52.8%	65.7%	32.1%	29.0%	NR	62.1%
Censored rate at one year of NPT	44.6%	NR	37.0%	NR	NR	31.1%

NR, not reported; ART, assisted reproductive technologies; NPT, NaProTechnology.

### 4.1 Main findings and cohort characteristics

This study presents the clinical outcomes of NPT in a cohort of 1,310 infertile couples, treated over a five-year period in a multidisciplinary center led by gynecologists specifically trained in NPT. To our knowledge, this is the largest series published to date on NPT. Its principal finding was a crude THB rate of 35.3%, increasing to 62.1% when adjusted for the duration of active participation in the NPT program.

The median age of women in our cohort falls within the range reported (34.0–37.2 years), while the median duration of infertility is at the lower end of the observed spectrum (24–67 months). Prior ART attempts were recorded in more than one quarter of participants, except for the series of 403 patients reported by Boyle et al. (17) which included only patients with a history of infertility and prior IVF. The relatively low awareness of NPT may explain the high proportion of couples with prior ART procedures. Among the 360 couples with a history of ART, the THB rate was 25.3%, lower than the 39.2% observed in patients without, but higher than the 17.0% cumulative spontaneous pregnancy rate reported in a retrospective cohort study of 1,320 couples who had been unsuccessfully treated by IVF and were followed for 7–9 years after the start of the treatment (21). This still represents a meaningful outcome that supports the potential benefit of offering NPT to these couples.

### 4.2 Etiological diagnosis in NPT: a distinctive clinical advantage

The notably high diagnostic success rate observed in our cohort reflects the comprehensive and individualized nature of the NPT evaluation process. This is particularly noteworthy when compared to reported data from ART centers, which often follow evaluation guidelines from organizations such as the American Society for Reproductive Medicine. According to these protocols, up to one in three cases may remain unexplained (22, 23). The ability to identify specific etiological factors in nearly all couples underscores a key advantage of NPT: its emphasis on detailed cycle tracking, targeted hormonal profiling, and multidisciplinary diagnostic workups., which prioritizes targeted medical and surgical interventions aimed at correcting specific health issues that may impair fertility.

### 4.3 Impact of surgery in NPT

Surgical fertility restoration procedures, previously common and effective (24), were abandoned due to widespread adoption of ART since the 1990s. This shift was partly due to the invasiveness of laparotomy-based techniques. Advances in endoscopic and robotic surgery have mitigated previous limitations and facilitated their resurgence.

In our series, nearly one-third of patients required surgical intervention, most commonly hysteroscopy or hysteroscopy combined with laparoscopy. These procedures were performed following a thorough diagnostic workup and were aimed at correcting structural or inflammatory abnormalities identified during the NPT evaluation. The role of hysteroscopy in infertility remains a subject of ongoing debate, as highlighted by Gulisano et al. (25), who emphasize both its diagnostic precision and therapeutic potential in selected cases. Within the NPT framework, hysteroscopy is not used as a routine screening tool but rather as a targeted intervention based on individualized cycle analysis and imaging findings. This distribution aligns with the multicenter experience reported by Stanford et al. (20) across five clinical sites. Most patients reported symptom improvement, and one in four achieved a viable pregnancy.

These findings highlight the role of restorative surgery as a key component of comprehensive fertility care within the NPT framework. However, the limited availability of experienced surgeons remains a significant barrier, often necessitating referral to other specialized centers for surgical management. This may lead to delays in NPT treatment and logistical challenges for patients and providers.

### 4.4 Determinants of pregnancy outcomes

Crude pregnancy rates reported in the literature show considerable variability. Our crude rate of 35.3% is slightly lower than the 38.0% reported by Tham et al. (18) in a cohort of 108 patients, and notably higher than the rates of 17.8%, 18.5%, and 25.5% reported in other studies (14, 17, 22). The higher rate in Tham's cohort (18) may be attributed to the high prevalence of patients with recurrent pregnancy loss. When adjusted for follow-up duration, pregnancy rates increased across all studies. The highest adjusted rate was 65.7% in Tham's study (18), followed closely by 62.1% in our cohort. These findings suggest that crude rates may underestimate the true effectiveness of the interventions when follow-up duration is not considered.

Maternal age remains a well-established determinant of pregnancy success. Our findings are consistent with this trend: adjusted pregnancy rates declined progressively with increasing age—87.3% in women under 30 years, 63.2% for ages 30–35, 53.3% for 36–40, and 24.4% for those over 40. These results align with previous studies, including Stanford et al. (14), which reported adjusted rates ranging from 59.1% in women under 30% to 46.1% in those aged 35–40 after 24 months of NPT. While these patterns are expected, the magnitude of decline observed in our study reinforces the importance of early intervention and individualized reproductive care strategies.

Previous studies have also reported differences in pregnancy rates according to the type of infertility, with higher rates typically observed in cases of secondary infertility compared to primary infertility (19). However, in our cohort, no significant differences were found between these groups. This suggests that, under the conditions of our study, the type of infertility may not be a decisive factor in predicting pregnancy outcomes. Importantly, our analysis identified recurrent pregnancy loss as an independent factor associated with a favorable outcome following NPT, as demonstrated in the logistic regression model. This finding highlights the potential of NPT in addressing reproductive challenges beyond conventional infertility classifications.

### 4.5 Study limitations

The retrospective, single-center, and non-randomized design limits causal inference between the interventions performed and the outcomes observed. This methodological limitation must be considered when interpreting the results, as it precludes direct comparison of NPT effectiveness with other strategies. Thus, the findings should be regarded as observational evidence from a real-world clinical setting—valuable for hypothesis generation and guiding future research, but insufficient to support definitive clinical conclusions. Although statistical models were applied to adjust for confounding factors, the lack of random allocation limits the ability to fully control for the influence of unmeasured variables.

A high withdrawal rate at 12 months (37.0%–48.1%) has been reported across all NPT (14, 17) studies, representing another important methodological limitation. Although the reasons for dropouts in NPT have not been formally evaluated, the principles of NPT support continuing treatment for at least 12–18 months after achieving satisfactory cycles. However, this recommended duration should not be interpreted as a fixed or mandatory timeframe for all couples. In our series, many presented with complex clinical profiles or had experienced prior failed attempts at other centers, making continuation particularly challenging. Other patients lacked a clear understanding of the nature and demands of NPT or were advised to discontinue treatment based on clinical judgment. This attrition impacts the reliability of the adjusted rate derived from life tables and its comparison with the crude rate. Kaplan–Meier analysis assumes non-informative censoring. However, if couples who discontinued treatment did so due to poor prognoses, this assumption may be violated, potentially leading to an overestimation of the adjusted THB rate. A sensitivity analysis modeled three scenarios: (A) 50% lower pregnancy probability among dropouts, (B) equal probability, and (C) 50% higher. Resulting adjusted cumulative pregnancy rates were 47.7%, 62.1%, and 76.5%, respectively, highlighting the impact of dropout assumptions.

Similar dropout rates (45%–69%) have been reported for ART, even in settings without financial restrictions on access (26, 27). Psychological burden, emotional distress, and poor prognosis have been identified as key factors contributing *to* withdrawal from IVF treatments (28, 29). Competing-risks methods such as the Fine–Gray model are well-suited to account for ART transitions, but our retrospective dataset and the limited granularity of dropout reasons precluded their application. We also acknowledge this as a methodological limitation and propose it as a direction for future prospective studies.

Additionally, population heterogeneity —such as infertility duration and, type, or prior ART exposure— complicates the identification of subgroups most likely to benefit from NPT. While this reflects real-world clinical practice, the lack of standardized inclusion criteria and limited documentation regarding reasons for exclusion may introduce selection bias. Future studies should include stratified analyses and clearer eligibility protocols to address this issue.

Finally, although multivariate analysis was performed to identify independent predictors of pregnancy, the variable selection process was just based on clinical judgment and univariate significance.

### 4.6 Clinical implications

The findings of this study suggest that NPT may offer a promising option for selected couples experiencing infertility. However, given the retrospective nature of the study and the lack of a control group, these results should be interpreted with caution. While the adjusted THB rate is encouraging, further prospective research is needed to confirm the clinical effectiveness of NPT in broader populations.

Several factors in this cohort posed additional challenges, including a mean infertility duration twice the recommended threshold for initiating NPT (12 months), relatively advanced maternal age (35 years), and a high proportion of patients with previous unsuccessful ART attempts. A substantial proportion of couples were simultaneously on the waiting list for ART within the public healthcare system. Many others opted to pursue NPT following multiple unsuccessful treatment attempts at other centers, in some cases despite having only a limited indication for NPT. Moreover, a learning curve among healthcare professionals is expected during the implementation of any novel approach, which may have influenced early outcomes in this cohort.

Overall, these results reflect the observed clinical effectiveness of NPT in real-world conditions, but they must be interpreted with caution due to the inherent limitations of the study design. No direct causal relationship between the intervention and outcomes can be established, nor can its efficacy be compared with other techniques such as IVF or IUI. Consequently, well-designed prospective, multicenter, and controlled studies are needed to validate these findings and support stronger clinical recommendations.

### 4.7 Future research directions

Prospective, ideally randomized, controlled studies would be desirable to directly compare NPT with other therapeutic strategies in homogeneous populations. However, such designs may not be ethically feasible in this context. Future research should aim to refine patient selection criteria, optimize surgical protocols, and explore long-term outcomes of NPT beyond pregnancy rates, including maternal health and child development.

Although the restorative nature of NPT may suggest potential economic advantages—such as reduced reliance on repeated ART cycles or avoidance of iatrogenic risks—no formal cost-effectiveness analysis was conducted in this study. In the absence of explicit cost inputs, including cost-per-THB or incremental cost-effectiveness ratios, any economic interpretation remains speculative. Future research should incorporate detailed cost data to enable robust economic evaluations and to better understand the financial implications of NPT within fertility care frameworks.

## 5 Conclusion

The initial experience from a pioneering center implementing a comprehensive NPT approach shows that this technology can achieve a high THB rate, when adjusted for the duration of active participation. These findings reflect the real-world clinical effectiveness of NPT, as they include all couples who sought consultation for infertility, regardless of diagnostic category, thus representing typical clinical practice. Although the results are promising, they should be considered preliminary and not conclusive. Large multicenter studies are needed to validate these findings, particularly to enable a direct comparison between NPT and other infertility treatment modalities. Further research is warranted to identify clinical predictors of NPT success, assess its psychological impact, investigate reasons for treatment discontinuation, and develop strategies to reduce early withdrawal.

## Data Availability

The raw data supporting the conclusions of this article will be made available by the authors, without undue reservation.
